# Cellular Crosstalk at the Gut–Liver Interface in Liver Cirrhosis: Epithelial Barrier Failure, Bacterial Translocation and Immune Activation as a Bidirectional Network

**DOI:** 10.3390/cells15191739

**Published:** 2026-09-23

**Authors:** Thomas N. Nitsotolis, Stelios F. Assimakopoulos, Eleftheria Mastoridou, Nikolaos-Gavriel Kolios, Dimitrios Biros, Eleni-Dimitra Oikonomidi, Maria Lagadinou, Alexia Papalexandrou, Haralampos Milionis, Eirini Christaki, Georgios Kalambokis

**Affiliations:** 1 1st Department of Internal Medicine and Infectious Diseases, Faculty of Medicine, School of Health Sciences, University of Ioannina, University Campus, 45110 Ioannina, Greece; e.mastoridou@uoi.gr (E.M.); ngkolios99@gmail.com (N.-G.K.); dimitrisbiros@gmail.com (D.B.); oikonomidieleni8@gmail.com (E.-D.O.); alexiapapalexandrou@gmail.com (A.P.); hmilioni@uoi.gr (H.M.); eirini.christaki@uoi.gr (E.C.); gkalambo@uoi.gr (G.K.); 2 Department of Internal Medicine, Medical School, University of Patras, 26504 Patras, Greece; sassim@upatras.gr (S.F.A.); m_lagad2004@yahoo.gr (M.L.)

**Keywords:** intestinal epithelial barrier, bacterial translocation, cellular crosstalk, gut–liver axis, cirrhosis-associated immune dysfunction, mucosal immunity, gut–vascular barrier, intestinal dysbiosis, systemic inflammation, acute-on-chronic liver failure

## Abstract

The gut–liver axis is central to the systemic complications of liver cirrhosis, but it is usually described as a linear cascade in which barrier failure releases microbial products that activate a downstream immune response. This review proposes instead a four-compartment framework, in which the epithelial/barrier, innate immune, adaptive effector, and stromal–endothelial compartments—the last including the gut–vascular barrier—are treated as reciprocally interacting nodes, and pathological bacterial translocation is treated as both an input to and an output of the network rather than as its endpoint. Applying this framework across the stages of cirrhosis, we examine how tight-junction disassembly, mucus-layer thinning, Paneth-cell dysfunction and reduced secretory IgA, together with dysbiosis and small-intestinal bacterial overgrowth, permit translocation; how translocated bacteria and pathogen-associated molecular patterns activate all four compartments; and how the resulting cytokine circuits further degrade the barrier, generating feed-forward loops that underlie cirrhosis-associated immune dysfunction and chronic systemic inflammation. We relate these circuits to spontaneous bacterial peritonitis, hepatic encephalopathy, aggravated portal hypertension, and acute-on-chronic liver failure, distinguishing throughout between mechanisms demonstrated in humans and those inferred from animal or in vitro models. We identified evidence through structured searches of PubMed/MEDLINE, Embase, and Web of Science, and report it according to the SANRA checklist. Framed as a network, biomarkers become compartment-specific reporters, and barrier-, microbiome-, and immune-directed therapies become interventions at defined nodes and edges—a view that clarifies both their rationale and the current limits of the evidence.

## 1. Introduction

Liver cirrhosis, the common histological end-stage of virtually all chronic liver diseases, remains a leading cause of global morbidity and mortality, accounting for roughly 2.4% of all deaths worldwide in 2019, with a rising contribution from metabolic dysfunction-associated and alcohol-related liver disease [1]. The natural history of cirrhosis is characterized by a shift from a compensated stage to a decompensated stage marked by the development of portal hypertension complications such as ascites, variceal hemorrhage, and hepatic encephalopathy. Acute-on-chronic liver failure (ACLF) may occur in patients with decompensated cirrhosis, increasing short-term mortality significantly [2]. Progressive breakdown of the gut–liver interaction is strongly linked to decompensation [3].

The anatomical and functional relationship between the gut and the liver, termed the “gut–liver axis”, provides the rationale for a gut-focused perspective of cirrhosis-related complications. The liver receives approximately 70% of its blood supply from the portal vein and is therefore continuously exposed to microbial products, metabolites, and antigens absorbed from the intestine, while returning bile acids, antimicrobial molecules, and immunoglobulins that shape the intestinal environment [3]. This interface is not a passive filter but a densely cellular, bidirectionally signaling structure. It is helpful to consider it as four interrelated compartments: the intestinal epithelial/barrier compartment; the resident and recruited innate immune cells of the lamina propria and liver; the adaptive effector lymphocytes; and the stromal–endothelial compartment, which includes subepithelial myofibroblasts and the gut–vascular barrier that gates entry into the portal circulation [3,4]. In health, continuous crosstalk among these compartments restricts microbial exposure to a physiological, tolerogenic minimum [5,6].

In cirrhosis, structural and functional failure of the epithelial barrier, along with intestinal dysbiosis and altered mucosal and systemic immune responses, collectively enable pathological bacterial translocation—the passage of viable bacteria and microbial products across the gut barrier to the mesenteric lymph nodes and beyond [7,8]. Translocation is commonly depicted as the terminal step of a one-way cascade, with immune activation as its downstream consequence. However, experimental findings from cirrhotic models indicate that mucosal immune dysregulation and stromal–vascular remodeling are major drivers of barrier failure and translocation, suggesting that translocation may serve both as an input to and an output of the cellular network involved; we note that this inference rests on experimental cirrhosis, and that human studies to date establish association rather than causal direction [4,9]. The resulting, self-amplifying engagement of innate and adaptive immunity sustains the systemic inflammation that characterizes cirrhosis-associated immune dysfunction (CAID) and triggers the major clinical complications of the disease [10,11].

Although researchers have extensively investigated the individual elements of this process—dysbiosis, barrier failure, translocation, and systemic inflammation—they often consider them in isolation and in a fixed upstream-to-downstream order. This narrative review integrates them into a single, clinician-oriented framework centered on reciprocal cellular crosstalk. After outlining the healthy multicellular interface, we examine its disruption in cirrhosis—perturbed microbial input, epithelial barrier collapse, and bidirectional bacterial translocation—then analyze the reciprocal epithelial, innate, adaptive, and stromal–endothelial circuits that convert local crosstalk into systemic inflammation. We then translate this network view into biomarkers that report on individual compartments, the clinical syndromes of decompensation, and barrier-, microbiome-, and immune-directed therapies conceived as interventions on interacting cell populations rather than on a single pathway.

### Scope and Relationship to Existing Reviews

The gut–liver axis in cirrhosis has been reviewed repeatedly from several angles, and a compartment-oriented reading is not new. Wiest and colleagues set out the translocation process itself [7]; Albillos and colleagues traced mechanism to therapy across chronic liver disease [3], and later described cirrhosis-associated immune dysfunction as an immune-compartment phenomenon [12]; Trebicka and colleagues organized the field around the microbiota and the events of decompensation [11]; Tranah and colleagues organized it around intervention [13]; Muñoz and colleagues described the shaping of gut immunity in cirrhosis compartment by compartment [14]; De Muynck and colleagues applied a single-lineage cellular lens to the same axis in this journal [15]; and, most recently, Yoon and colleagues have mapped the cellular architecture and molecular mediators of gut–liver–immune crosstalk [16], and Olaru-Stavila and colleagues have described barrier failure in cirrhosis layer by layer [17]. Table 1 summarizes these accounts and their organizing principles.

Against that background, the present review is distinguished less by the use of compartments than by four choices. First, it is cirrhosis-specific and stage-resolved: the same four-compartment logic is applied to the compensated, decompensated, and acute-on-chronic stages in turn, whereas broader accounts span all chronic liver disease [3,18] and etiology-specific ones do not cross stages. Second, bidirectionality is the organizing axis rather than a closing observation; the principal existing accounts run in one direction—from gut to liver [7,11] or from liver to immune system [12]—and the contribution attempted here is to close that loop explicitly, to identify which arm of it rests on which kind of evidence, and to treat translocation as an output of the network as well as an input to it. Third, the review integrates the human evidence published between 2024 and 2026 that post-dates most of these accounts: single-nucleus sequencing of cirrhotic terminal ileum [19], the identification of perivascular macrophages as a checkpoint at the gut–vascular barrier [20], and the behavior of barrier and gut–vascular markers across disease stages [21]. Fourth, every mechanistic claim is labeled by provenance—human, animal, in vitro, or inferred—in the text and in Supplementary Table S5, allowing the reader to see where the network as described is demonstrated and where it is extrapolated. This review does not attempt to be a review of gut-directed therapy, for which Tranah and colleagues [13] and Bloom and Chung [22] are the appropriate sources, nor a review of microbiota composition, for which Trebicka and colleagues [11] and Chang and colleagues [23] are; therapeutic and microbiological material appears here only insofar as it bears on the cellular network.

## 2. Materials and Methods: Evidence Identification and Selection

### 2.1. Search Strategy

We searched PubMed/MEDLINE, Embase (Ovid) and Web of Science Core Collection from database inception to 31 August 2026, combining terms for the interface (“intestinal barrier”, “tight junction”, “intestinal permeability”, “mucus layer”, “Paneth cell”, “secretory IgA”, “gut–vascular barrier”), for the microbial input (“gut microbiome”, “dysbiosis”, “small intestinal bacterial overgrowth”, “bacterial translocation”, “endotoxemia”, “lipopolysaccharide”), for the cellular compartments (“mucosal immunity”, “innate lymphoid cells”, “Th17”, “regulatory T cells”, “mucosa-associated invariant T cells”, “Kupffer cell”, “enteric glia”, “cellular crosstalk”), and for the clinical context (“liver cirrhosis”, “portal hypertension”, “spontaneous bacterial peritonitis”, “cirrhosis-associated immune dysfunction”, “acute-on-chronic liver failure”, “hepatic encephalopathy”, “hepatocellular carcinoma”), together with intervention terms (“rifaximin”, “norfloxacin”, “probiotics”, “synbiotics”, “fecal microbiota transplantation”, “farnesoid X receptor”, “non-selective beta-blockers”, “albumin”, “sodium-glucose cotransporter-2 inhibitors”). Supplementary Table S6 provides the complete search strings for each database. We hand-searched the reference lists of included articles and major reviews, and searched ClinicalTrials.gov and the WHO International Clinical Trials Registry Platform for registered but unpublished trials.

### 2.2. Eligibility

We included English-language studies reporting original data on the intestinal barrier, the gut microbiota, bacterial translocation, or mucosal and systemic immune activation in adults with liver cirrhosis, as well as mechanistic studies in established animal or in vitro models of cirrhosis or cholestasis, and clinical practice guidelines from major hepatology societies. We excluded case reports, narrative reviews used as primary data sources, studies confined to non-cirrhotic liver disease unless they addressed a mechanism with no cirrhosis-specific counterpart, and studies in which cirrhosis could not be distinguished from earlier disease stages.

### 2.3. Prioritization of Evidence

Evidence was prioritized in a fixed order: first, randomized controlled trials and their meta-analyses; second, prospective human cohorts and mechanistic studies performed on human tissue; third, retrospective human cohorts; and fourth, animal and in vitro studies, which were used to supply mechanisms where human data are absent and are labeled as such in the text and in Supplementary Table S5. Where randomized and observational evidence conflicted, we presented both and stated the conflict rather than resolving it. We used review articles only to provide conceptual framing and did not cite them as sources of primary data. Where a finding is available only as a conference abstract or a preprint, this is stated explicitly at the point of citation.

### 2.4. Reporting

Reporting follows the SANRA (Scale for the Assessment of Narrative Review Articles) checklist, which is provided as Supplementary File S1. We note that SANRA addresses reporting transparency rather than search completeness. As a narrative review, this work is not registered, records were not screened in duplicate, and a formal count of the records retrieved, screened, and excluded at each stage was not maintained; accordingly, no flow diagram of study selection is presented, and the review does not claim the reproducibility of a systematic review. These limitations are restated in Section 12.

## 3. The Gut–Liver Interface as a Multicellular Network in Health

The intestinal barrier is not a single structure but a set of concentric, functionally integrated layers maintained by continuous dialog among distinct cell populations; together they permit nutrient absorption while restricting microbial access to the internal milieu [3,24]. Viewing these layers as four interacting cellular compartments—epithelial/barrier, innate immune, adaptive immune, and stromal–endothelial—provides the framework used throughout this review and is summarized for the healthy gut in Figure 1.

The epithelial/barrier compartment begins with the commensal microbiota, which resists pathogen colonization through competition for nutrients and niches, production of bacteriocins and short-chain fatty acids, and maintenance of a low luminal redox potential [3]. Overlying the epithelium, a chemical layer of mucus—organized as a firm inner and a looser outer stratum in the colon and dominated by the MUC2 mucin—physically separates luminal bacteria from the epithelial surface; Paneth cells at the base of the small-intestinal crypts secrete α-defensins, lysozyme, and C-type lectins such as the regenerating islet-derived protein 3 (Reg3) family, which shape microbial composition and enforce the spatial segregation of bacteria from the mucosa [3,25]. The physical barrier is formed by a single layer of columnar epithelial cells sealed by the apical junctional complex; tight junctions—composed of occludin and the claudin family coupled to the actin cytoskeleton through the zonula occludens (ZO) scaffolding proteins—regulate paracellular permeability and define the selectivity of the barrier [24,26]. Critically, the epithelium is not a passive wall: intestinal epithelial cells sample the lumen through pattern-recognition receptors, transport secretory immunoglobulin A (sIgA) to coat and neutralize commensals, and instruct the underlying immune system through alarmins including thymic stromal lymphopoietin, IL-25 and IL-33, thereby setting the tone of mucosal immunity [5,6].

Beneath the epithelium, the innate compartment of the lamina propria enforces tolerance to commensals while remaining poised to respond to invaders. Tissue-resident macrophages and dendritic cells sample luminal antigens, clear the low levels of bacteria that constitutively cross the epithelium, and calibrate the threshold for inflammation [3]. Group 3 innate lymphoid cells respond to myeloid IL-23 and IL-1β by secreting IL-22, which acts back on the epithelium to reinforce antimicrobial peptide production and junctional integrity, a prototypical homeostatic feedback loop between the innate and epithelial compartments [27]. After absorption into the portal blood, Kupffer cells continue this innate surveillance in the liver, positioning the hepatic macrophage pool as the distal node of the same circuit [3,11]. The adaptive compartment translates microbial cues into durable, antigen-specific regulation: gut-associated lymphoid tissue and lamina propria plasma cells generate the sIgA that confines bacteria to the lumen, while the microbiota calibrates the balance between pro-inflammatory T-helper-17 (Th17) cells and regulatory T cells [28], and mucosa-associated invariant T cells add a layer of rapid, innate-like antibacterial responsiveness [10].

The fourth compartment is structural and vascular. Subepithelial myofibroblasts, pericytes, and enteric glia support the epithelium and modulate local inflammatory and reparative signaling, while the endothelium of the intestinal microvasculature forms the gut–vascular barrier. Analogous in organization to the blood–brain barrier—endothelial cells ensheathed by pericytes and enteric glial cells, with junctional regulation dependent on Wnt/β-catenin signaling—the gut–vascular barrier controls whether bacteria and their products that breach the epithelium can reach the portal circulation and disseminate systemically [4]. It thus constitutes the final cellular checkpoint between the gut and the liver, and recent single-cell and human biopsy work has identified intestinal blood-vessel-associated macrophages as regulators of this checkpoint [20]. In health, these four compartments operate as interlocking feedback loops rather than a linear chain, and host-derived signals—notably bile acids acting through the nuclear farnesoid X receptor (FXR) to induce enteroprotective and antimicrobial genes and restrain small-intestinal bacterial overgrowth—help synchronize them [25]. The remainder of this review examines how cirrhosis dismantles this reciprocal crosstalk, converting mutually stabilizing circuits into mutually amplifying ones.

## 4. Intestinal Dysbiosis and Small-Intestinal Bacterial Overgrowth: The Perturbed Microbial Input

The microbial layer is the first compartment to change in cirrhosis, and its reorganization is inseparable from the barrier and immune abnormalities that follow [3,11]. In a landmark case–control metagenomic study, Qin and colleagues identified tens of thousands of microbial genes that distinguished patients with cirrhosis from healthy controls, with notable enrichment of buccal (oral)-origin taxa in the gut [29]. At the taxonomic level, the cirrhotic microbiome is characterized by the expansion of potentially pathogenic, predominantly Gram-negative and oral-origin taxa—including Enterobacteriaceae (notably *Escherichia coli* and *Klebsiella* species), Streptococcaceae, Enterococcaceae, and Veillonellaceae—with depletion of beneficial autochthonous commensals such as Lachnospiraceae, Ruminococcaceae, and *Faecalibacterium prausnitzii* [29,30]. Bajaj and colleagues distilled these opposing shifts into the Cirrhosis Dysbiosis Ratio, which decreases progressively from healthy controls through compensated to decompensated and hospitalized patients, correlates inversely with endotoxemia, and predicts organ failure and death [30]. Contemporary metagenomic and multi-omic cohorts have extended these observations, linking oral–gut microbial overlap and its antimicrobial-resistance gene burden to disease severity [31] and, in longitudinal analyses, tracking microbial and metabolic change with clinical course [32]; microbiome composition is further shaped by etiology, with alcohol in particular disrupting the microbiota, the mucus and epithelial barrier, and antimicrobial peptide production [33].

Superimposed on this qualitative dysbiosis is a quantitative abnormality: small-intestinal bacterial overgrowth (SIBO). Impaired intestinal motility with prolonged transit, reduced gastric acidity—often aggravated by proton-pump-inhibitor use—and diminished bile flow make overgrowth substantially more common in cirrhosis than in health [34]. Meta-analyses give a pooled prevalence of approximately 41% against 11% in controls, with an odds ratio close to 6.8 [35], and an updated synthesis of 34 case–control studies gives 36.9% in compensated and 42.9% in decompensated disease, rising to 57.7% in the presence of spontaneous bacterial peritonitis [36]. Individual estimates, however, range from roughly 30% to 60%, and this spread reflects ascertainment as much as biology. Against jejunal aspirate culture as the reference standard, the glucose hydrogen breath test has a pooled sensitivity of only 54.5% (specificity 83.2%) and the lactulose breath test 42.0% (specificity 70.6%), with substantial between-study heterogeneity and further variation according to the diagnostic cut-off [37]; the glucose breath test performs particularly poorly in cirrhosis [38]. Prevalence figures derived from breath testing and from aspirate culture are therefore not directly comparable, and the wide confidence intervals in the pooled estimates are better read as a measure of methodological heterogeneity than of true biological variability [39]. What is consistent across methods is the direction and the clinical associations: overgrowth is more frequent in decompensated disease and is associated with ascites, minimal hepatic encephalopathy, bacterial translocation and spontaneous bacterial peritonitis (Table S2).

## 5. Breakdown of the Epithelial Barrier Compartment in Cirrhosis

Against this altered microbial input, the epithelial barrier compartment fails at every level, converting the tightly regulated gut–liver interface into a conduit for microbes and their products (Table S1) [3,24]. A defining structural abnormality is the disruption of epithelial tight junctions. In duodenal biopsies from patients with cirrhosis, Assimakopoulos and colleagues demonstrated a significant reduction in the expression of occludin and claudin-1 compared with healthy controls; the loss was more pronounced in decompensated than in compensated disease, correlated inversely with the Child–Pugh score and the grade of esophageal varices, and paralleled the degree of systemic endotoxemia [26]. Direct physiological confirmation now exists: in ex vivo studies of duodenal biopsies, transepithelial electrical resistance was lower in compensated cirrhosis than in controls (13.3 versus 18.9 Ω·cm^2^), establishing that barrier failure is present already at the compensated stage [40]. Single-nucleus RNA sequencing of human terminal ileum has since resolved these changes to the cellular level, showing that stem cells, enterocytes and, most markedly, Paneth cells are progressively depleted with advancing decompensation. Defensin, mucin, and mucus-sulfation genes are downregulated, although the small sample size of that study warrants caution [19]. Reduced α-defensin output has also been shown directly in the cirrhotic intestinal mucosa [41].

Several interacting processes drive this barrier failure. Portal hypertension causes congestion and edema of the intestinal wall—portal-hypertensive enteropathy—with structural changes that impair barrier function [24]. Intestinal oxidative stress promotes enterocyte apoptosis, modulates tight-junction proteins, and impairs brush-border function [42]. The mucus layer thins, and antimicrobial peptide and Paneth-cell defenses weaken, eroding the chemical barrier [3]. Reduced delivery of bile acids to the gut removes a key FXR-dependent brake on bacterial overgrowth and mucosal injury; in experimental cholestasis, FXR agonism with obeticholic acid restored ileal barrier function and reduced bacterial translocation, underscoring the causal contribution of impaired bile-acid signaling [25,43]. Finally, the deeper gut–vascular barrier is disrupted: in experimental cirrhosis, its remodeling opens additional entry sites for bacterial translocation, and FXR controls this checkpoint [4,44]. The net functional consequence is a measurable increase in intestinal permeability that tracks with disease severity and predicts complications, and the injured epithelium actively signals to the other compartments even as it loses its physical integrity [6,7,24].

## 6. Pathological Bacterial Translocation: Input to and Output of the Network

Bacterial translocation is defined as the passage of viable bacteria or their products from the intestinal lumen across the mucosal barrier to the mesenteric lymph nodes and, potentially, the systemic circulation [7,8]. Some translocation to the mesenteric lymph nodes occurs physiologically and is immunologically contained; pathological translocation denotes an excessive, dysregulated process—driven by the confluence of barrier failure, dysbiosis/SIBO, and immune paresis—that overwhelms these containment mechanisms in cirrhosis [7]. Mechanistically, translocation proceeds by paracellular passage through disrupted tight junctions and by transcellular routes, delivering not only viable bacteria but also a spectrum of pathogen-associated molecular patterns (PAMPs)—lipopolysaccharide, bacterial DNA, peptidoglycan and muramyl dipeptide—into the lamina propria, the mesenteric lymph nodes and the portal and systemic circulations [7], while disruption of the deeper gut–vascular barrier removes a further checkpoint [4].

Direct human evidence of translocation comes from detecting bacterial products in normally sterile compartments. Using PCR-based methods, Such and colleagues detected bacterial DNA simultaneously in blood and culture-negative, non-neutrocytic ascitic fluid in approximately one-third of patients with cirrhosis, with *Escherichia coli* the most frequently identified organism, and sequence identity between blood and ascites indicating single-clone translocation episodes [45]. More recent work confirms and extends this: in a prospective cohort of 294 patients without infection, serum bacterial DNA was detected in 36.1% and independently predicted 6-month mortality (hazard ratio 2.49, 95% CI 1.22–5.11) [46]. The predominance of Gram-negative enteric organisms among translocating bacteria reflects the dysbiotic expansion of Enterobacteriaceae described above [47].

Although translocation is conventionally placed at the end of a one-way cascade, experimental evidence indicates it also results from the cellular responses it provokes. In rats with CCl4-induced cirrhosis, dysbiosis-driven dysregulation of the intestinal immune system tracks with disease stage and contributes to barrier impairment and bacterial translocation, indicating that mucosal immune activation is not merely a consequence of translocation but also a driver of it [9]. In human cirrhosis, the corresponding evidence is associative: barrier, translocation and immune-activation markers covary with each other and with disease stage, and no human study has established the direction of the relationship. Translocation therefore serves, on present evidence, simultaneously as an input to and an output of the epithelial–immune–stromal network, an overview of which, from health to decompensation, is shown in Figure 2 [11,48].

## 7. Cellular Crosstalk Across the Four Compartments in Cirrhosis

The events described so far—perturbed microbial input, epithelial barrier collapse, and bidirectional translocation—converge on reciprocal cell–cell signaling that determines the clinical behavior of cirrhosis [10]. Rather than a one-way relay from microbe to cytokine, translocated bacteria and PAMPs set in motion interlocking circuits among the four compartments; Figure 3 summarizes the principal cells, mediators, feedback loops, and the type of evidence supporting each, and Table S5 lists them.

### 7.1. Epithelial, Innate and Adaptive Circuits

The epithelial compartment initiates and shapes the response rather than merely yielding to it. Through pattern-recognition receptors—chiefly Toll-like receptor 4 for lipopolysaccharide and nucleotide-binding oligomerization domain-like receptors—intestinal epithelial cells signal through MyD88- and TRIF-dependent pathways to activate nuclear factor-κB and prime the inflammasome; injured enterocytes also secrete an instructive repertoire of alarmins and chemokines that program the underlying compartments [5,6,7]. This alarmin biology is established in murine and human intestinal models and inferred for cirrhosis. A dense network of innate cells receives these signals and translocated PAMPs: lamina propria macrophages and dendritic cells, and, after portal delivery, hepatic Kupffer cells release tumor necrosis factor-α, interleukin-6, and interleukin-1β [10]. In contrast, group 3 innate lymphoid cells secrete IL-22 in a reparative feedback that becomes insufficient as disease advances—a circuit demonstrated in murine models [27]. Circulating monocytes shed soluble CD14 and presepsin upon lipopolysaccharide engagement, and hepatic LBP rises, providing measurable readouts of innate activation [49]. Because the same portal blood that delivers microbial products also perfuses the liver, the intestinal and hepatic myeloid compartments function as a single epithelial–myeloid–Kupffer axis [3,12].

The adaptive compartment both responds to and reshapes this environment. The dysbiotic microbiota biases CD4+ differentiation towards Th17 cells at the expense of regulatory T cells, increasing mucosal IL-17 and IL-22 output [28]; in human cirrhosis, elevated IL-17 has been documented in circulating innate-like and conventional T cells [50], and IL-17F tracks infection-independent progression to acute-on-chronic liver failure [51]. Impaired generation and transepithelial delivery of secretory IgA weakens microbial confinement—fecal IgA and IgA-coated bacteria are altered in cirrhosis and correlate with severity [52], and a hepatic pIgR-dependent mechanism links IgA secretion to the control of translocation in mice with human tissue validation [53]—while mucosa-associated invariant T cells are chronically engaged and undergo pyroptosis that scales with translocation markers in patients [54]. Adaptive effector cytokines act back upon the epithelium: IL-22 and IL-17 drive repair at physiological levels but, when sustained, contribute to injury and further barrier compromise, closing a loop between the adaptive and epithelial compartments [6].

### 7.2. The Stromal–Endothelial Checkpoint, Feed-Forward Loops and Cirrhosis-Associated Immune Dysfunction

Stromal and vascular cells between the mucosa and portal blood gate the reach of these responses. Subepithelial myofibroblasts and enteric glia modulate local signaling—a supportive role characterized largely in non-cirrhotic models—whereas the gut–vascular barrier normally determines whether microbes and PAMPs that breach the epithelium can enter the portal circulation [4]. In experimental cirrhosis, this deeper checkpoint is disrupted, with remodeling that opens additional entry sites [4,44], and human evidence now implicates intestinal blood-vessel-associated macrophages, whose depletion promotes translocation independently of liver disease and whose dysfunction is evident in patient duodenal biopsies [20]. Taken together, these circuits are reciprocal and self-amplifying: epithelial alarmins recruit innate cells; innate IL-23 and antigen presentation polarize adaptive effectors; adaptive and innate cytokines feed back to degrade the barrier and disrupt the gut–vascular checkpoint; and the resulting increase in translocation re-enters the network at the top. In experimental cirrhosis, immune activation thus drives barrier failure, not only its consequence, though whether the same holds in human disease has not been tested directly [4,9]. These events unfold on the background of cirrhosis-associated immune dysfunction (CAID)—the coexistence of systemic inflammation and immunodeficiency in which impaired hepatic clearance and reduced synthesis of pattern-recognition and complement proteins compromise bacterial killing. At the same time, persistent exposure to gut-derived PAMPs sustains low-grade inflammation [10,12]. Scaled to the whole organism, this crosstalk manifests as the systemic inflammation that large European cohorts identify as the hallmark of decompensated cirrhosis and the principal driver of acute-on-chronic liver failure [55], operating through nitric oxide-mediated splanchnic vasodilation, immune-mediated tissue injury and a reallocation of metabolic fuel [11,48]. Contemporary syntheses place these circuits at the center of infection susceptibility [56]; portal hypertension and systemic inflammation now interact throughout progression rather than in sequence [57], and prospective human data show that portal decompression by transjugular intrahepatic portosystemic shunt reverses markers of gut-barrier and immune dysfunction [58].

## 8. Biomarkers of Barrier Failure, Translocation and Immune Activation

The crosstalk described above can be interrogated clinically through circulating and fecal biomarkers that report on distinct nodes of the network (Table 2; an extended matrix is provided in Table S3) [24]. To keep mechanistic interest separate from clinical utility, each marker is assigned a biomarker class following the FDA–NIH BEST (Biomarkers, EndpointS, and other Tools) resource—diagnostic, monitoring, prognostic, or predictive—with the additional designation “mechanistic” for markers whose present value is that they report on a defined compartment rather than that they inform a clinical decision [59]. Clinical readiness is described in three levels. No single marker is specific for gut-derived translocation, and each must be interpreted within the individual clinical context.

Markers of microbial exposure and monocyte activation are the best characterized. Lipopolysaccharide-binding protein (LBP) is elevated in patients with cirrhosis and ascites, particularly those with the most marked immune and hemodynamic derangement, and falls with intestinal decontamination [49]; it is best read as a mechanistic and monitoring marker confounded by hepatic synthetic capacity. Soluble CD14 and presepsin index monocyte activation, and presepsin independently predicts short-term mortality [60]. Markers of the barrier itself—intestinal fatty-acid-binding protein (I-FABP) and tight-junction-associated proteins such as zonula occludens-1—reflect enterocyte injury [61], while fecal calprotectin—a diagnostic biomarker in the BEST sense whose evaluated context of use is screening rather than confirmation—flags patients who warrant assessment for hepatic encephalopathy and spontaneous bacterial peritonitis, which continue to be diagnosed by clinical criteria and by the ascitic-fluid neutrophil count, respectively [62]. Bacterial DNA in blood or ascites is mechanistic and, based on recent evidence, prognostic for 6-month mortality, but it is not diagnostic of infection and does not direct treatment [45,46]; soluble urokinase plasminogen activator receptor (suPAR) reflects generalized immune activation and predicts short-term mortality [63]. A recent head-to-head comparison across stages of cirrhosis found that plasmalemma vesicle-associated protein, a marker of gut–vascular barrier damage, predicted transplant-free survival, whereas zonulin-family peptides discriminated poorly—a reminder that these markers are not interchangeable and that none is confounder-free [21]. Benchmarked against a systematic review of prognostic biomarkers in decompensated cirrhosis, in which interleukin-6, urinary NGAL and copeptin each added prognostic information to MELD [64], no marker of gut-derived translocation yet has an established assay standard, a cirrhosis-specific cut-off or a demonstrated predictive role in matching a patient to a gut-directed therapy.

## 9. Clinical Consequences of the Gut–Liver Crosstalk

The reciprocal crosstalk described above is not merely a mechanistic curiosity; it is the proximate cause of the syndromes that define decompensated cirrhosis and determine its prognosis.

### 9.1. Infection and Spontaneous Bacterial Peritonitis

Spontaneous bacterial peritonitis (SBP)—an infection of previously sterile ascitic fluid in the absence of a surgically treatable source—is a classic paradigm of translocation-related infection, arising when translocating enteric organisms seed the ascites [7]. Consistent with the dysbiotic growth of Enterobacteriaceae, Gram-negative enteric bacteria have historically dominated, although a rising share of Gram-positive and multidrug-resistant (MDR) organisms now complicates management [65]. In a worldwide prospective study, the global prevalence of MDR bacteria among patients with cirrhosis and bacterial infections was approximately 34%, independently associated with poorer resolution, more organ failures, and higher mortality [65], and European data corroborate this trend [66]. Notably, a substantial share of infections in cirrhosis arise at urinary, respiratory, and skin sites rather than in the abdomen [67], underscoring that not all infections in cirrhosis are gut-derived. Because infections often precipitate further decompensation, antibiotic prophylaxis in selected high-risk patients and prompt empirical therapy are fundamental [68].

### 9.2. Portal Hypertension, Systemic Hemodynamics and Hepatic Encephalopathy

Translocation-driven inflammation aggravates the circulatory abnormalities of cirrhosis. Gut-derived endotoxins and the subsequent cytokine cascade promote nitric oxide and other vasodilators, accentuating splanchnic arterial vasodilation and effective arterial hypovolemia [48]; patients with the highest LBP levels exhibit the greatest immune and hemodynamic disturbance, which improves with intestinal decontamination [49]. Increased permeability and translocation markers correlate with portal hypertension severity and improve with non-selective beta-blockers, independent of hemodynamic response, suggesting that barrier disruption may drive decompensation [69]. Systemic inflammation increases progressively before the first decompensating event [70], and the current consensus model incorporates this inflammatory dimension [71]. Hepatic encephalopathy (HE) exemplifies the interaction between gut-derived toxins and systemic inflammation: although ammonia of largely intestinal origin is the conventional trigger, its correlation with clinical grade is inconsistent, and systemic inflammation and infection sensitize the brain to ammonia [72]. Translocation-driven inflammation thus modulates the onset and severity of HE, supporting gut-directed therapy; the poorly absorbed antibiotic rifaximin lowers the risk of recurrent HE and HE-related hospitalization [73].

### 9.3. Acute-on-Chronic Liver Failure

Acute-on-chronic liver failure (ACLF), a syndrome of acute decompensation with organ failure and high short-term mortality as defined by the CANONIC study, is distinguished by intense systemic inflammation [74]. Because systemic inflammation is the principal driver, its sources are potential therapeutic targets. The gut is one of several, and the evidence does not support treating it as the predominant one. In the PREDICT cohort of 1273 patients with acute decompensation, proven bacterial infection precipitated 29.4% of episodes in patients who went on to develop ACLF and severe alcohol-related hepatitis in a further 26.6%, while no precipitant was identified in 50.9%; the corresponding figure for stable decompensated cirrhosis was 66.0% [75]. The EASL clinical practice guidelines likewise note that systematic workup fails to identify a precipitant in 35% of patients with ACLF [76]. Severe alcohol-related hepatitis acts principally through sterile hepatocyte injury and the release of damage-associated molecular patterns, and a substantial proportion of precipitating infections arise at non-gut sites [67]. Gut-derived PAMPs are therefore one important input among several into a final common pathway of systemic inflammation, which the systemic-inflammation hypothesis holds causes organ failure through vasodilatation, immunopathology, and metabolic reprioritization—none of which requires a gut-derived trigger as its proximate cause [48]. What distinguishes gut-derived signals is not that they predominate, but that they are, in principle, modifiable; current guidance on ACLF incorporates the interplay of systemic inflammation and organ failure [77].

The same circuits may also operate over a longer timescale. Chronic, low-grade engagement of the pattern-recognition pathways described above has been implicated in hepatocarcinogenesis. In chronically injured mouse liver, gut sterilization or Toll-like receptor 4 inactivation reduces tumor burden by acting on promotion rather than initiation [78], and microbiota-mediated bile-acid metabolism regulates hepatic accumulation of CXCR6+ natural killer T cells and, hence, liver-selective immunosurveillance [79]. In patients, a distinct gut-microbiota profile with increased permeability and systemic inflammation accompanies hepatocellular carcinoma in metabolic dysfunction-associated cirrhosis [80], and a microbiota signature has been shown to precede clinical diagnosis [81]. This evidence establishes temporality and biological plausibility, but not causation, and no completed trial has shown that modulating the microbiota alters the incidence or the outcome of hepatocellular carcinoma [82]. Sustained translocation and dysbiosis are therefore best described as candidate contributors whose modifiability is untested, and this remains the least mature clinical extension of the network described here [83].

## 10. Therapeutic Implications: Targeting Cellular Crosstalk

Because the crosstalk network can be modulated at multiple nodes, established and emerging interventions target the gut–liver axis, and each can be associated with the compartment it predominantly modulates (Table 3, Figure 4; extended evidence is given in Table S4) [3]. It is useful to distinguish interventions that act directly on the gut lumen and mucosa from those that act indirectly on the network through portal pressure, systemic hemodynamics, or innate immune and endothelial output. This grouping is a heuristic rather than a strict dichotomy—bile-acid-directed agents, in particular, act on both the luminal bile-acid pool and hepatic and systemic signaling—but it clarifies where each intervention enters the cascade.

### 10.1. Interventions Acting Directly on the Gut Lumen and Mucosa

#### 10.1.1. Poorly Absorbed Antibiotics and Selective Decontamination

Antibiotic strategies that reduce the luminal microbial load are the best-established gut-directed therapies. Rifaximin maintains remission from HE and reduces HE-related hospitalization [73] and is a key component of current guidelines [84]; a mechanistic randomized trial also showed that it reduces gut-derived inflammation and mucin degradation and lowers infection risk, although it did not meet its primary endpoint on neutrophil oxidative burst [85]. Its benefit does not extend to every setting—a randomized trial in severe cirrhosis with ascites was null [86]—but it prevents overt HE after transjugular intrahepatic portosystemic shunt [87]. Primary norfloxacin prophylaxis reduces SBP, delays hepatorenal syndrome and improves survival in high-risk patients [88], though use must be restricted to guideline-defined indications because of MDR [68]; reassuringly, rifaximin exposure was not associated with drug-resistant infection in a large inpatient cohort [89].

#### 10.1.2. Fecal Microbiota Transplantation

Restoring the microbial input directly is the most-studied microbiome-directed strategy, and the trials are informative about the network beyond their clinical endpoints. In the first randomized trial, an enema from a rationally selected donor was safe and reduced further episodes of HE [90]; a phase 1 capsule trial without antibiotic pretreatment is the most mechanistically informative, raising duodenal E-cadherin and α-defensin 5, lowering duodenal IL-6 and lowering serum LBP—that is, acting on the barrier and translocation limbs of the axis and not only on ammonia [91]—with benefit persisting at 12 to 15 months [92] and a fall in gut antimicrobial-resistance genes [93]. The largest randomized experience, the THEMATIC trial, allocated 60 patients across four arms varying route, dose, and donor diet; transplantation was safe, and HE recurred in 9% of those receiving any active transplant against 40% of those receiving placebo alone (odds ratio 0.15, 95% CI 0.04–0.64), an efficacy signal the investigators report as post hoc [94]. Engraftment depended on recipient Lachnospiraceae abundance rather than on the delivery variable, and the accompanying multi-omic analysis linked recurrence to short-chain-fatty-acid and secondary-bile-acid metabolism [95]. In alcohol-associated disease, the endpoints are harder: transplantation outperformed prednisolone for 90-day survival, principally by averting infection deaths [96], dampened the interleukin-1β response in alcohol-associated ACLF [97], and produced an early survival benefit in a meta-analysis that attenuated by six months [98]. The evidence is mixed: a defined eight-strain consortium did not meet its efficacy endpoint, with only two strains engrafting in most recipients [99], and a network meta-analysis of 17 trials ranked synbiotics, not transplantation, first for reducing blood ammonia [100]. Outside recurrent *Clostridioides difficile* infection, the intervention remains investigational [101], the most explicit formal endorsement being a 2025 expert consensus [102]; two adequately powered trials with hard endpoints—one with an infection endpoint [103], one with a decompensation-or-death endpoint [104]—and a trial interrogating the peritoneal compartment directly [105] are now running. Results reported so far only as a congress abstract and preprint (the PROFIT trial) are cited as such [106].

#### 10.1.3. Probiotics, Defined Consortia and Bile-Acid-Directed Agents

Probiotics and synbiotics have reduced translocation markers and improved HE in some studies, although heterogeneity limits firm conclusions [3]. Restoring bile-acid signaling addresses an upstream driver of dysbiosis and barrier failure: FXR activation induces intestinal antibacterial defenses [25], and in experimental cholestasis the FXR agonist obeticholic acid restored ileal barrier function and reduced translocation [43], though this avenue remains preclinical. Novel strategies that neutralize or adsorb microbial products are in early development; the carbon-nanoporous adsorbent Yaq-001 met a safety and tolerability endpoint in a 28-patient randomized trial, with mechanistic signals on permeability and inflammation [107].

### 10.2. Interventions Acting on the Network Indirectly

#### 10.2.1. Non-Selective Beta-Blockers, Albumin, and Portal Decompression

Beyond lowering portal pressure, non-selective beta-blockers reduce intestinal permeability and translocation, lowering LBP and interleukin-6 even in hemodynamic non-responders [69], an effect with a mechanistic basis in experimental cirrhosis [108]. Human albumin has immunomodulatory and endothelial-stabilizing properties beyond volume expansion, and long-term administration prolonged survival as a disease-modifying treatment in the ANSWER trial [109]. Portal decompression by transjugular intrahepatic portosystemic shunt reverses markers of gut-barrier and immune dysfunction, suggesting the loop is partly reversible from the hemodynamic end [58]. Adequate nutrition and avoiding unnecessary acid suppression, which promotes SIBO, are complementary measures [34].

#### 10.2.2. Sodium–Glucose Cotransporter-2 Inhibitors

Interest in this class arises from renal physiology rather than from the gut [110]. Sodium and water retention in cirrhosis depends substantially on augmented proximal tubular sodium reabsorption mediated by the apical sodium–hydrogen exchanger 3; because that exchanger and the cotransporter occupy a shared apical microdomain, selective cotransporter inhibition suppresses exchanger activity, increases sodium chloride delivery to the macula densa, and restores tubuloglomerular feedback with downstream modulation of the renin–angiotensin–aldosterone system [111]. The clinical evidence in ascites is early and mixed: a meta-analysis of three studies in 382 patients found higher complete ascites resolution (odds ratio 2.39) and weight loss of 4.86 kg but concluded that current data preclude routine use [112]; a pilot study by two of the present authors reported increased natriuresis and improved circulatory and cardiac function in refractory ascites [113]; and the natriuretic effect is transient, present at day 3 and absent by day 28 [114]. No society guideline, including the 2025 practice update on ascites, mentions the class [115]. The point of contact with the network described here is the infection endpoint, and the evidence there is contradictory: a propensity-matched cohort of 10,660 patients reported lower hazards of SBP (0.55) and hepatorenal syndrome (0.47) [116], whereas the only placebo-controlled randomized trial reported more infection (55% versus 20%) and acute kidney injury (50% versus 15%) [117], and a large cohort in compensated cirrhosis found lower hazards of decompensation and death [118]. Crucially, no study has measured intestinal permeability, endotoxemia, or bacterial translocation under this class in cirrhosis; the evidence that these agents restore tight-junction proteins and reduce lipopolysaccharide translocation is preclinical [119], and the two human studies connecting the class to the gut in liver disease are in metabolic dysfunction-associated steatotic liver disease rather than cirrhosis [120,121]. These agents are therefore best regarded as adjacent, hypothesis-generating interventions rather than an established node of gut–liver crosstalk [122].

### 10.3. A Network View and Combination Strategies

Viewed through the lens of cellular crosstalk, the interventions above act on defined nodes and edges of the epithelial–immune–stromal network [13]. Microbiome-directed strategies reshape the microbial input; bile-acid- and barrier-directed agents act on the epithelial compartment; non-selective beta-blockers limit the microbial signal reaching the innate and stromal–vascular compartments; and albumin and immunomodulatory measures act on the systemic output. Framing therapy in these terms generates a testable hypothesis rather than a conclusion: that simultaneously targeting two compartments might outperform single-node intervention, and that compartment-specific biomarkers might match treatment to the dominant abnormality in an individual patient. Neither proposition has been tested. The only combination trial to report, of simvastatin with rifaximin in 237 patients, did not meet its primary endpoint—ACLF developed in 17.9% of treated patients against 14.2% of controls (hazard ratio 1.23, 95% CI 0.65–2.34) [123]. That result does not refute the hypothesis, since neither agent was selected on a compartment-specific biomarker, but it establishes that combination alone is not sufficient. We therefore present the network view as a framework for designing such studies rather than as a therapeutic recommendation.

## 11. Conclusions

Barrier failure and pathological bacterial translocation link the gut to the immune and clinical complications of cirrhosis. However, the relationship is reciprocal rather than linear: the epithelial, innate immune, adaptive immune and stromal–endothelial compartments form interlocking circuits in which translocation is as much an output of immune and stromal–vascular activation as an input to it. Whether this framing changes practice depends on five open questions. First, human cellular resolution of these circuits remains rudimentary; the single-cell and spatial datasets available from cirrhotic intestine are very small, and the best-characterized reciprocal arms are those demonstrated in rodents. Second, no barrier or translocation biomarker has an established assay standard, a cirrhosis-specific threshold, or evidence that acting on it improves outcome. Third, no cohort has quantified the relative contribution of gut-derived microbial products to systemic inflammation versus sterile hepatocyte injury and non-intestinal infection. Fourth, microbiome-directed therapy has not yet translated into hard outcome benefit, and two adequately powered trials with infection and decompensation endpoints will report before 2029. Fifth, whether targeting two compartments simultaneously outperforms targeting one remains untested. Answering these questions, rather than adding mechanisms, is what would make a network view of the gut–liver interface clinically consequential.

### Clinical Practice Points

1. Consider translocation-driven systemic inflammation as a contributor whenever a patient with cirrhosis decompensates without an obvious precipitant.
2. Use rifaximin, with lactulose, to prevent recurrent hepatic encephalopathy.
3. Offer antibiotic prophylaxis for spontaneous bacterial peritonitis only within guideline-defined high-risk indications, mindful of multidrug resistance.
4. Recognize that non-selective beta-blockers have gut-barrier and translocation-limiting effects beyond portal-pressure reduction.
5. Consider long-term albumin as a disease-modifying therapy in decompensated cirrhosis with ascites.
6. Avoid unnecessary proton-pump-inhibitor use, which promotes small-intestinal bacterial overgrowth.
7. Interpret barrier and translocation biomarkers as compartment-specific reporters, within the full clinical context, and recognize that none is yet validated to guide the choice of a gut-directed therapy.

## 12. Limitations

This narrative review has several limitations. First, as a non-systematic review, it is not registered; the search—although structured and dated—was not performed in duplicate, the number of records retrieved and excluded at each stage was not formally recorded, and no formal risk-of-bias assessment was undertaken, so selection bias cannot be excluded; we prioritized randomized controlled trials, large cohorts and human mechanistic studies, and followed the SANRA checklist, which addresses the transparency of reporting rather than the completeness of the search. Second, gut microbiome signatures in cirrhosis show substantial cohort-to-cohort and geographical variability and are confounded by etiology and diet, so interpret specific dysbiotic patterns cautiously [29,30]. Third, much of the mechanistic evidence for reciprocal crosstalk—including the reparative innate-lymphoid-cell and stromal arms and FXR-dependent barrier repair—derives from animal and in vitro systems, and its cellular resolution in human cirrhosis is only beginning to emerge [9]; the provenance of each mechanism is labeled in Supplementary Table S5. Fourth, several biomarkers lack assay standardization and cirrhosis-specific cut-offs. Finally, most barrier-directed and compartment-targeted therapies beyond established antibiotics and albumin remain investigational, and their effect on hard clinical endpoints awaits confirmation.

## Figures and Tables

**Figure 1:**
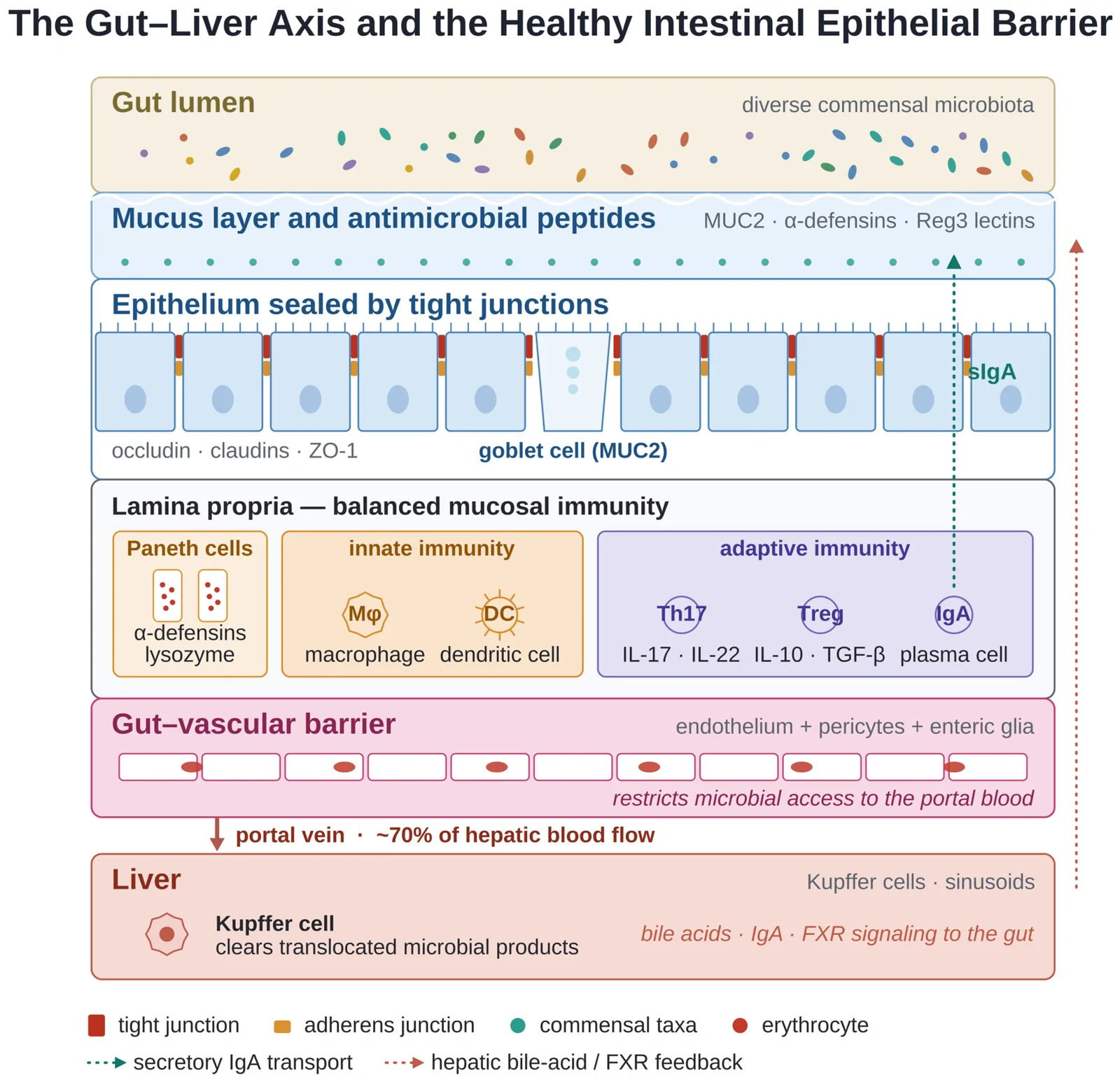
The gut–liver axis and the healthy intestinal epithelial barrier. Successive, functionally integrated layers—the luminal commensal microbiota; the mucus and antimicrobial-peptide layer (MUC2, Paneth-cell α-defensins, Reg3 lectins); the epithelium sealed by tight junctions (occludin, claudins, zonula occludens-1); the balanced mucosal immunity of the lamina propria; and the gut–vascular barrier—restrict microbial access to the portal circulation, which supplies approximately 70% of hepatic blood flow. Colored circles denote diverse commensal taxa. sIgA, secretory immunoglobulin A; FXR, farnesoid X receptor. This figure is an original work of the authors; no third-party material was reproduced or adapted. An artificial-intelligence assistant was used solely for graphical and aesthetic refinement of author-designed content, and the authors conceived and verified the scientific content.

**Figure 2:**
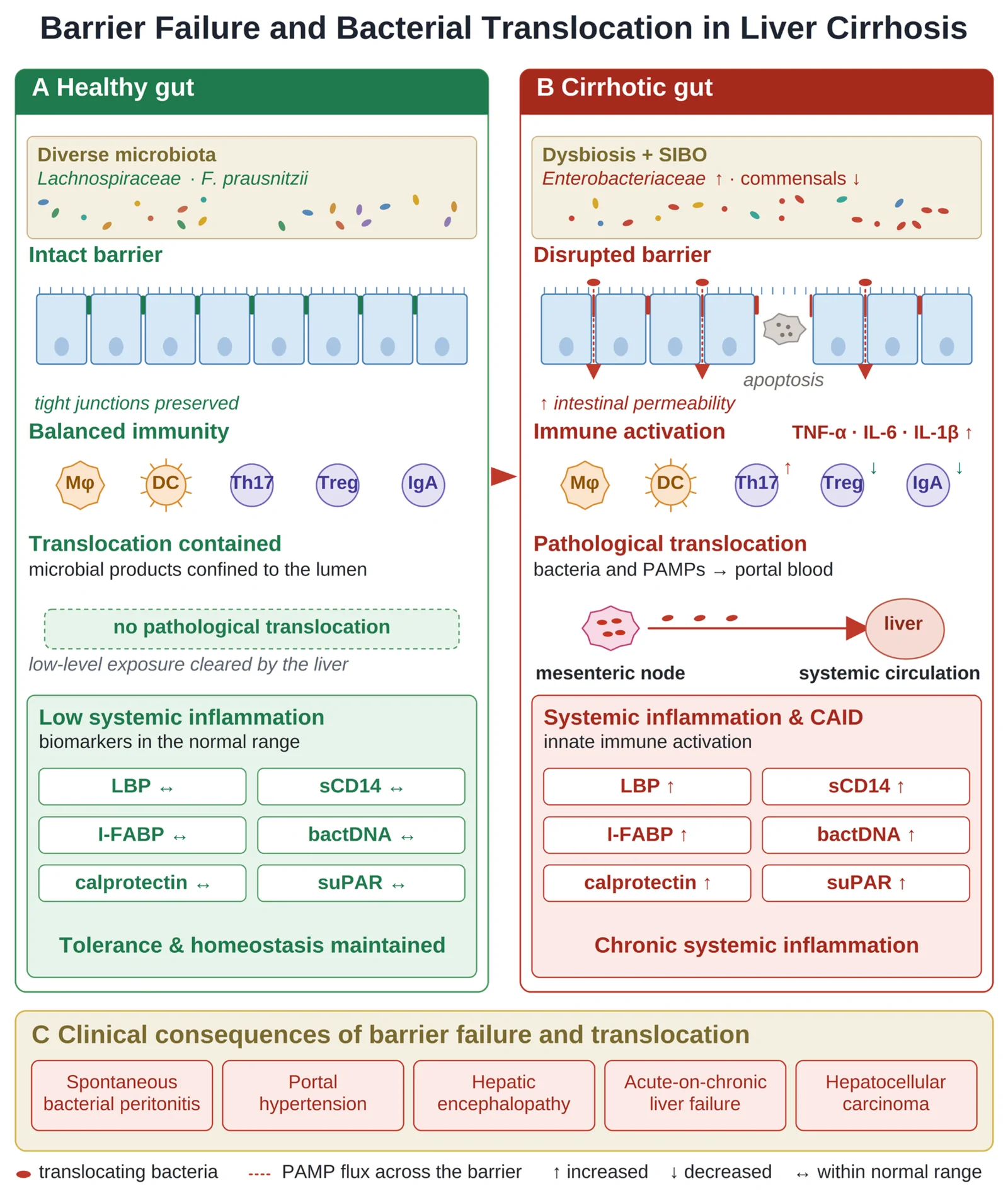
Barrier failure and pathological bacterial translocation in liver cirrhosis. (**A**) The healthy gut maintains a diverse microbiome, an intact epithelial barrier, balanced mucosal immunity, and low systemic inflammation, with barrier and translocation biomarkers within the normal range. (**B**) In cirrhosis, dysbiosis and small-intestinal bacterial overgrowth, tight-junction disruption, and enterocyte injury permit pathological translocation of bacteria and pathogen-associated molecular patterns to the mesenteric lymph nodes and systemic circulation, driving systemic inflammation and cirrhosis-associated immune dysfunction (CAID) with elevated biomarkers. (**C**) These processes precipitate the major clinical consequences of decompensated cirrhosis. ↑ increased; ↓ decreased; ↔ within normal range. This figure is an original work of the authors; no third-party material was reproduced or adapted. An artificial-intelligence assistant was used solely for graphical and aesthetic refinement of author-designed content, and the authors conceived and verified the scientific content.

**Figure 3:**
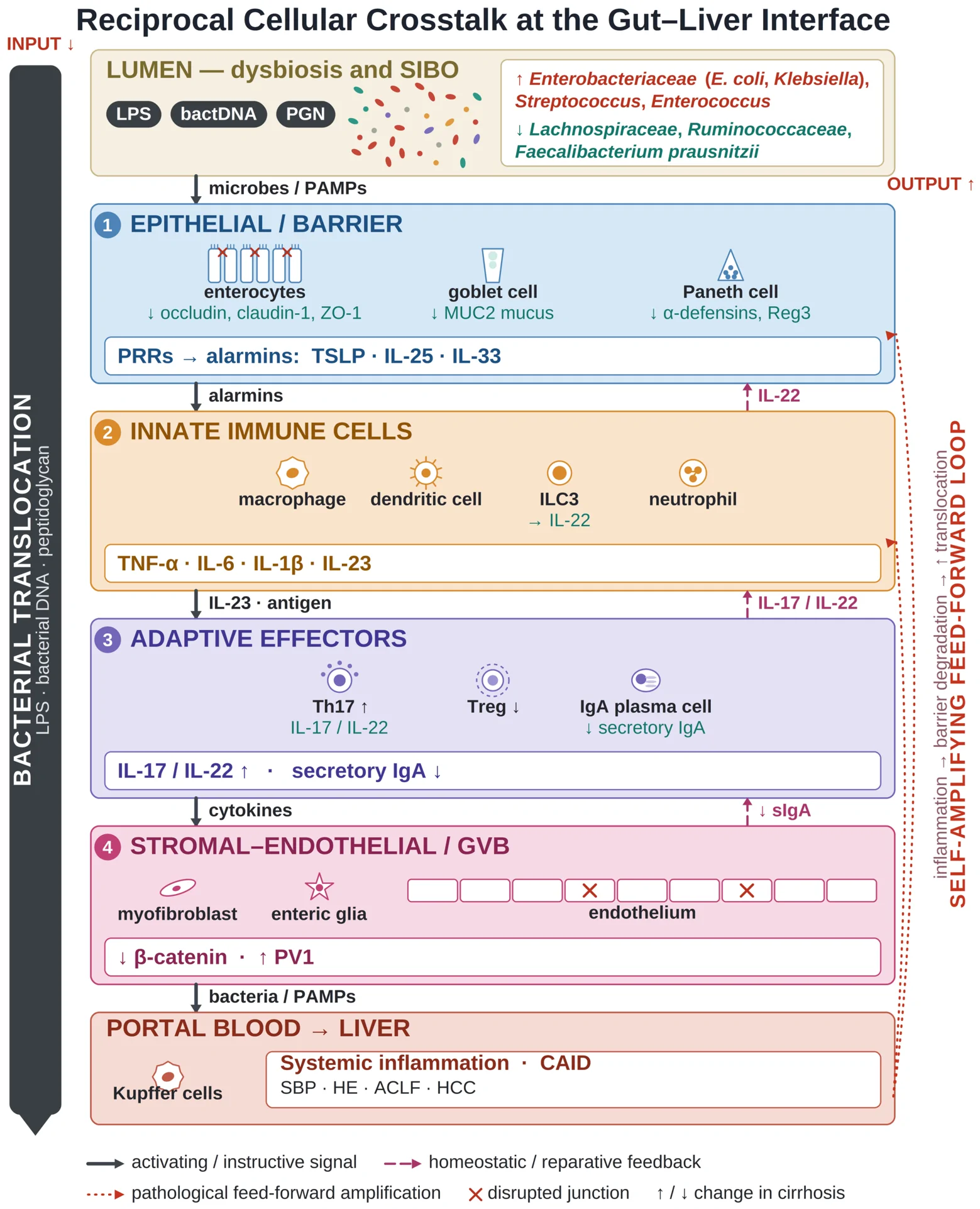
Reciprocal cellular crosstalk at the gut–liver interface in cirrhosis. The interface is depicted as four interacting cellular compartments and their bidirectional signaling. (**1**) The epithelial/barrier compartment senses luminal microbes and pathogen-associated molecular patterns (PAMPs) through pattern-recognition receptors and, as barrier integrity is lost, releases alarmins (TSLP, IL-25, IL-33), chemokines, and Reg3 lectins. (**2**) Innate immune cells respond with TNF-α, IL-6, IL-1β and IL-23, while ILC3-derived IL-22 feeds back onto the epithelium. (**3**) Adaptive effectors show a shift towards Th17 at the expense of regulatory T cells (Treg), with IL-17/IL-22 output and impaired secretory IgA. (**4**) The stromal–endothelial compartment and the gut–vascular barrier (GVB) gate systemic dissemination; GVB disruption permits entry of bacteria and PAMPs into the portal circulation. Arrow styles distinguish activating (solid), homeostatic-feedback (long-dashed), and pathological feed-forward (dotted) signals. Bacterial translocation is both an input to and an output of these circuits, generating self-amplifying loops that scale to cirrhosis-associated immune dysfunction (CAID) and systemic inflammation. sIgA, secretory immunoglobulin A. This figure is an original work of the authors; no third-party material was reproduced or adapted. An artificial-intelligence assistant was used solely for graphical and aesthetic refinement of author-designed content, and the authors conceived and verified the scientific content.

**Figure 4:**
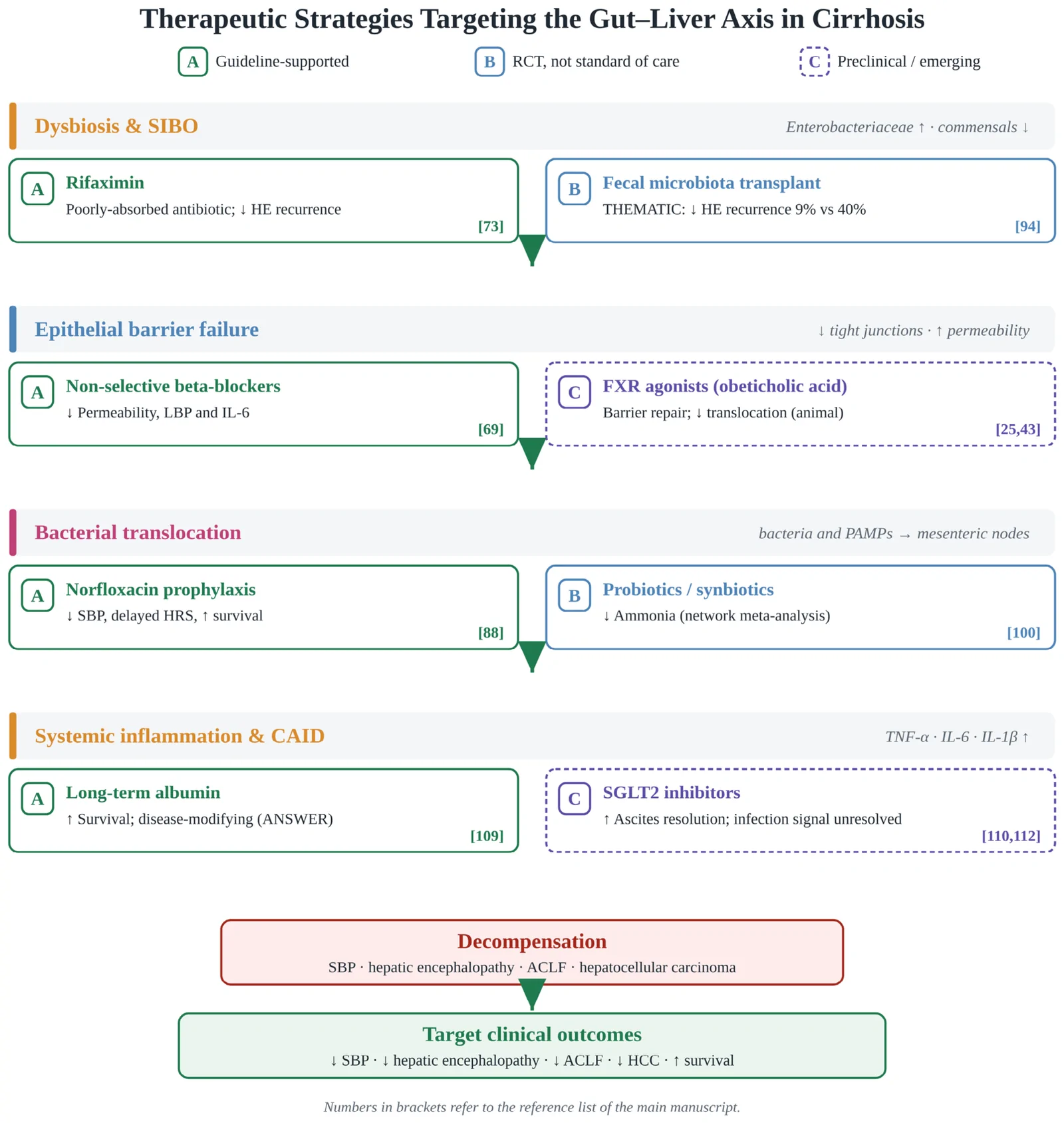
Therapeutic strategies targeting the gut–liver axis in cirrhosis. Interventions are mapped onto the principal compartments and interactions of the gut–liver network, from the perturbed microbial input through barrier failure and immune activation to decompensation. They are graded by level of evidence: A (guideline-supported), B (randomized evidence but not standard of care), and C (preclinical or emerging). ACLF, acute-on-chronic liver failure; FMT, fecal microbiota transplantation; HCC, hepatocellular carcinoma; HE, hepatic encephalopathy; HRS, hepatorenal syndrome; SBP, spontaneous bacterial peritonitis; SGLT2i, sodium–glucose cotransporter-2 inhibitors. This figure is an original work of the authors; no third-party material was reproduced or adapted. An artificial-intelligence assistant was used solely for graphical and aesthetic refinement of author-designed content, and the authors conceived and verified the scientific content.

**Table 1:** Narrative reviews of the gut–liver axis in cirrhosis and what the present four-compartment, bidirectional framework adds. Reference numbers, where shown, correspond to the reference list.

Review	Organizing Principle	Scope	Contribution of the Present Review
Wiest et al., 2014 [7]	The translocation process itself, step by step	Cirrhosis; gut → liver	Pre-dates the gut–vascular barrier and single-cell eras; compartments described as a cascade rather than reciprocally coupled nodes
Albillos et al., 2020 [3]	Mechanism → disease → therapy	All chronic liver disease	Not cirrhosis-specific and not stage-resolved; remains the standard reference for the therapeutic rationale
Tranah et al., 2021 [13]	Organized by intervention class	Cirrhosis; therapy-first	Therapeutic rather than mechanistic in organization; the present review is complementary and defers to it on treatment
Trebicka et al., 2021 [11]	Microbiota, organized by decompensation event	Cirrhosis; microbiome-centric	Centers the microbial input rather than the host cellular response; unidirectional in construction
Albillos et al., 2022 [12]	Immune compartments: inflammation vs. immunodeficiency	Cirrhosis-associated immune dysfunction	The liver → immune arm of the loop; the gut barrier enters as a source rather than as a reciprocally regulated compartment
Muñoz et al., 2023 [14]	Compartment and mechanism, gut-immunity-centric	Cirrhosis; mucosal immunity	Closest precedent for a compartment reading in cirrhosis; focused on gut immunity and predates the 2024–2026 human barrier datasets
De Muynck et al., 2021 [15]	A single cellular lineage (macrophages) across the axis	Chronic liver disease	Establishes the cell-centric approach in this journal; single-lineage rather than multi-compartment
Yoon et al., 2026 [16]	Immune-cell architecture across gut and liver, then by disease	Gut–liver–immune axis, multiple diseases	The closest current comparator; immune-architecture-led and disease-comparative rather than cirrhosis-specific, and does not calibrate evidence provenance

**Table 2:** Biomarkers of intestinal barrier dysfunction, bacterial translocation and immune activation in cirrhosis, classified by biomarker class and clinical readiness.

Biomarker	Compartment Reported	Class	Principal Evidence in Cirrhosis	Clinical Readiness
I-FABP (FABP2)	Enterocyte injury	Mechanistic	Released from damaged enterocytes; marker of barrier failure	Investigational
ZO-1	Tight-junction integrity	Mechanistic	Tracks barrier injury; prognostic value shown in bacteremic sepsis	Investigational
Zonulin-family peptides	Paracellular permeability (putative)	Mechanistic (cautionary)	Discriminated poorly across stages; assay specificity contested	Not recommended
PV-1 (PLVAP)	Gut–vascular barrier	Mechanistic; prognostic	Rises in decompensation without further rise in ACLF; predicts transplant-free survival	Investigational
LBP	Microbial exposure; hepatic acute-phase	Mechanistic; monitoring	Elevated in ascitic cirrhosis; falls with norfloxacin and beta-blockade	Investigational; confounded by hepatic synthesis
sCD14/presepsin	Monocyte activation	Prognostic	Presepsin predicts 28-day mortality	Validated in cohorts; not standardized
Bacterial DNA	Translocation, direct	Mechanistic; prognostic	In one third of culture-negative ascites; serum detection predicts 6-month mortality	Investigational; not diagnostic of infection
Fecal calprotectin	Intestinal inflammation	Diagnostic (screening context of use)	Rises with severity; evaluated as a screening tool for HE and SBP, not as a confirmatory test	In routine use for other indications
suPAR	Systemic immune activation	Prognostic	Compartmentally regulated; predicts short-term mortality	Validated in cohorts
Cirrhosis Dysbiosis Ratio	Microbial input	Mechanistic; prognostic	Falls with decompensation and infection; predicts organ failure and death	Research use only

Classes follow the FDA–NIH BEST resource; “mechanistic” is not a BEST category and is used for markers whose established value is that they report on a defined compartment of the gut–liver network. Screening is not a separate BEST class but a context of use of a diagnostic biomarker; fecal calprotectin is accordingly classed as diagnostic, with screening as its context of use, because it has been evaluated to identify patients who warrant confirmatory assessment for HE and SBP, not to confirm either diagnosis. No marker listed is predictive in the BEST sense. I-FABP, intestinal fatty-acid-binding protein; ZO-1, zonula occludens-1; PV-1, plasmalemma vesicle-associated protein; LBP, lipopolysaccharide-binding protein; sCD14, soluble CD14; suPAR, soluble urokinase plasminogen activator receptor; HE, hepatic encephalopathy; SBP, spontaneous bacterial peritonitis; ACLF, acute-on-chronic liver failure.

**Table 3:** Established and emerging interventions targeting the gut–liver axis in cirrhosis, grouped by direct versus indirect action and graded by level of evidence.

Intervention	Node Targeted	Setting	Key Finding	Level
**Acting directly on the gut lumen and mucosa**				
Rifaximin	Microbial input	Recurrent HE	↓ HE recurrence and HE-related hospitalization; ↓ gut-derived inflammation and mucin degradation; ↓ infection (OR 0.21)	A (HE); B (barrier)
Norfloxacin prophylaxis	Selective decontamination	Low-protein ascites	↓ SBP, delayed HRS, ↑ survival	A
Fecal microbiota transplantation	Microbial input; epithelial barrier	Recurrent HE	THEMATIC: HE recurrence 9% vs. 40% (OR 0.15); ↑ E-cadherin, DEFA5; ↓ IL-6, LBP; ↓ resistance genes	B
Fecal microbiota transplantation	Microbial input	Severe AH; alcohol ACLF	90-day survival 75% vs. 56.6% vs. prednisolone, driven by fewer infection deaths	B
Defined consortia (VE303)	Microbial input	HE	Primary efficacy endpoint not met; poor strain engraftment	C
Probiotics/synbiotics	Microbial input; translocation markers	HE; surrogate markers	Synbiotics ranked first for ammonia reduction (network meta-analysis)	B (surrogate)
Enterosorbent (Yaq-001)	Luminal PAMP load	Cirrhosis	Met safety/tolerability endpoint (n = 28); efficacy not tested	C
FXR agonists	Epithelial barrier; GVB	Experimental cholestasis	Restored ileal barrier; ↓ translocation (animal only)	C
**Acting on the network indirectly**				
Non-selective beta-blockers	Portal pressure; permeability	Portal hypertension	↓ Permeability, LBP, IL-6, partly independent of hemodynamic response	A (PHT); B (barrier)
Long-term albumin	Systemic innate response; endothelium	Decompensated cirrhosis + ascites	↑ Survival; disease-modifying (ANSWER)	A
TIPS	Portal pressure; barrier and immune dysfunction	Refractory ascites; bleeding	Reverses markers of barrier and immune dysfunction; I-FABP falls within 24 h	A (indication); B (barrier)
SGLT2 inhibitors	Renal Na handling; ascites	Cirrhotic ascites	↑ Ascites resolution (OR 2.39) but data preclude routine use; contradictory infection signals	C (emerging)
Simvastatin + rifaximin	Combined	Decompensated cirrhosis	Primary endpoint not met; ACLF 17.9% vs. 14.2% (HR 1.23)	B (null)

Evidence levels: A, supported by clinical practice guidelines and standard of care for the stated indication; B, supported by randomized trials but not yet standard of care, including surrogate-endpoint and informative null trials; C, preclinical or emerging. AH, alcohol-associated hepatitis; ACLF, acute-on-chronic liver failure; DEFA5, α-defensin 5; FXR, farnesoid X receptor; GVB, gut–vascular barrier; HE, hepatic encephalopathy; HRS, hepatorenal syndrome; LBP, lipopolysaccharide-binding protein; PAMP, pathogen-associated molecular pattern; PHT, portal hypertension; SBP, spontaneous bacterial peritonitis; SGLT2, sodium–glucose cotransporter-2; TIPS, transjugular intrahepatic portosystemic shunt.

## Data Availability

No new data were created or analyzed in this study. Data sharing is not applicable to this article.

## Supplementary Materials

The following supporting information can be downloaded at: https://www.mdpi.com/article/10.3390/cells15191739/s1, Table S1: components of the intestinal barrier and their alteration in cirrhosis; Table S2: gut-microbiota alterations and small-intestinal bacterial overgrowth in cirrhosis; Table S3: extended matrix of barrier, translocation and immune-activation biomarkers in cirrhosis; Table S4: detailed evidence for interventions targeting the gut–liver axis in cirrhosis; Table S5: principal cells and mediators of inter-compartment crosstalk with the type of supporting evidence; Table S6: full electronic search strings for each database; File S1: Completed SANRA checklist. Reference [124] is cited in the Supplementary File S1.

## Abbreviations

The following abbreviations are used in this manuscript: ACLF, acute-on-chronic liver failure; CAID, cirrhosis-associated immune dysfunction; FXR, farnesoid X receptor; GVB, gut–vascular barrier; HCC, hepatocellular carcinoma; HE, hepatic encephalopathy; I-FABP, intestinal fatty-acid-binding protein; IL, interleukin; ILC, innate lymphoid cell; LBP, lipopolysaccharide-binding protein; LPS, lipopolysaccharide; MAIT, mucosa-associated invariant T; MDR, multidrug-resistant; NSBB, non-selective beta-blocker; PAMP, pathogen-associated molecular pattern; PV-1, plasmalemma vesicle-associated protein; Reg3, regenerating islet-derived protein 3; SBP, spontaneous bacterial peritonitis; sCD14, soluble CD14; SGLT2, sodium–glucose cotransporter-2; SIBO, small-intestinal bacterial overgrowth; sIgA, secretory immunoglobulin A; suPAR, soluble urokinase plasminogen activator receptor; Th17, T-helper-17; TIPS, transjugular intrahepatic portosystemic shunt; TLR, Toll-like receptor; TNF-α, tumor necrosis factor-alpha; Treg, regulatory T cell; ZO-1, zonula occludens-1.
