# Supplementary material for: Cellular Crosstalk at the Gut–Liver Interface in Liver Cirrhosis: Epithelial Barrier Failure, Bacterial Translocation and Immune Activation as a Bidirectional Network
Source: Cells. 2026 Sep 23;15(19):1739. doi: 10.3390/cells15191739 (PMC13664048; doi:10.3390/cells15191739)
Supplement: Supplementary file 1 [file cells-15-01739-s001.zip › cells-4564769-supplementary.pdf]

Supplementary Materials

Cellular Crosstalk at the Gut–Liver Interface in Liver Cirrhosis: Epithelial Barrier Failure, Bacterial Translocation and Immune Activation as a Bidirectional Network

Thomas N. Nitsotolis, Stelios F. Assimakopoulos, Eleftheria Mastoridou, Nikolaos-Gavriel Kolios, Dimitrios Biros, Eleni-Dimitra Oikonomidi, Maria Lagadinou, Alexia Papalexandrou, Haralampos Milionis, Eirini Christaki and Georgios Kalambokis

Reference numbers in these tables correspond to the reference list of the revised main manuscript. Table S5 has been extended with an Evidence-type column, and Table S6 provides the full electronic search strings.

Table S1—components of the intestinal barrier and their alteration in cirrhosis.

Barrier layer	Principal cellular source/constituents	Function in health	Alteration in cirrhosis	Ref.
Microbiological (luminal microbiota)	Commensal bacteria (*Lachnospiraceae*, *Ruminococcaceae*, *Faecalibacterium prausnitzii*)	Colonization resistance through competition for nutrients and niches, bacteriocins, short-chain fatty acids, and a low luminal redox potential	Dysbiosis with expansion of *Enterobacteriaceae*, *Streptococcaceae*, *Enterococcaceae*, *Veillonellaceae* and oral-origin taxa; depletion of autochthonous commensals; superimposed small-intestinal bacterial overgrowth	[3,29,30,35]
Chemical – mucus	Goblet cells (MUC2 mucin)	Firm inner and looser outer mucus strata that physically separate luminal bacteria from the epithelial surface	Thinning of the mucus layer with loss of the spatial segregation of bacteria from the mucosa	[3]
Chemical – antimicrobial peptides	Paneth cells (α-defensins, lysozyme, Reg3 lectins)	Shaping of microbial composition and enforcement of bacterial–mucosal segregation	Paneth-cell dysfunction with reduced α-defensin and Reg3 output, aggravated by reduced intestinal bile-acid delivery and FXR signaling	[3,25]
Physical – apical junctional complex	Enterocytes (occludin, claudins, zonula occludens proteins)	Regulation of paracellular permeability and definition of barrier selectivity	Reduced occludin and claudin-1 expression in duodenal biopsies; more pronounced in decompensated disease; inversely correlated with Child–Pugh score and varices grade and paralleled by endotoxemia	[24,26]
Physical – epithelial monolayer	Single layer of columnar intestinal epithelial cells	Physical separation of lumen and lamina propria; luminal sampling through pattern-recognition receptors	Enterocyte apoptosis and brush-border dysfunction driven by intestinal oxidative stress and by portal-hypertensive enteropathy with mucosal congestion and edema	[24,42]
Immunological – secretory	Lamina propria plasma cells; epithelial transcytosis of secretory IgA	sIgA coats and neutralizes commensals and confines bacteria to the lumen	Impaired generation and transepithelial delivery of secretory IgA	[3,10,12]
Immunological – cellular	Lamina propria macrophages and dendritic cells, ILC3, Th17/Treg, MAIT cells	Tolerance to commensals with IL-22-dependent reinforcement of epithelial defense	Th17/Treg imbalance, sustained myeloid activation and reparative IL-22 output that becomes insufficient as disease advances	[9,27,28]
Vascular – gut–vascular barrier	Endothelium with pericytes and enteric glia; Wnt/β-catenin-dependent junctions	Final cellular checkpoint gating entry of microbes and their products into the portal circulation	Remodeling with reduced β-catenin signaling and increased PV1, opening additional entry sites; dysfunction of intestinal blood-vessel-associated macrophages	[4,20,44]

Abbreviations: FXR, farnesoid X receptor; ILC3, group 3 innate lymphoid cell; MAIT, mucosa-associated invariant T cell; PV1, plasmalemma vesicle-associated protein; Reg3, regenerating islet-derived protein 3; sIgA, secretory immunoglobulin A; Th17, T-helper-17 cell; Treg, regulatory T cell.

Table S2: Gut-microbiota alterations and small-intestinal bacterial overgrowth in cirrhosis.

Domain	Observation	Population/model	Clinical correlate	Ref.
Metagenomic signature	Tens of thousands of microbial genes discriminate cirrhosis from controls, with enrichment of taxa of buccal (oral) origin in the gut	Case–control gut metagenomics	Invasion of the intestinal ecosystem from the oral cavity	[29]
Taxonomic expansion	*Enterobacteriaceae* (*Escherichia coli*, *Klebsiella* spp.), *Streptococcaceae*, *Enterococcaceae*, *Veillonellaceae*	Cirrhosis cohorts	Provides the organisms that translocate and that predominate among spontaneous bacterial peritonitis isolates	[29,30,47]
Taxonomic depletion	*Lachnospiraceae*, *Ruminococcaceae*, *Faecalibacterium prausnitzii*	Cirrhosis cohorts	Loss of short-chain fatty-acid production and of colonization resistance	[29,30]
Cirrhosis Dysbiosis Ratio (CDR)	Ratio of autochthonous to non-autochthonous taxa falls stepwise from controls to compensated, decompensated, and hospitalized patients	Prospective cohort	Inverse correlation with endotoxemia; falls further with hepatic encephalopathy and infection; predicts organ failure and death	[30]
Etiology-specific effects	Alcohol disrupts the microbiota, the mucus and epithelial barrier, and antimicrobial peptide production	Alcohol-related liver disease	Amplified microbial exposure of the liver	[33]
SIBO – prevalence	Approximately 41% in cirrhosis versus 11% in controls (odds ratio ≈ 6.8), rising to about 50% in decompensated disease	Systematic review and meta-analysis	Associated with ascites, minimal hepatic encephalopathy, bacterial translocation and spontaneous bacterial peritonitis	[35]
SIBO – mechanism	Impaired intestinal motility with prolonged transit, reduced gastric acidity (often proton-pump-inhibitor related) and diminished bile flow	Clinical and experimental studies	Rationale for avoiding unnecessary acid suppression in cirrhosis	[34]
Bile-acid axis	Reduced delivery of bile acids to the gut removes the FXR-dependent restraint on bacterial overgrowth and mucosal injury	Experimental cholestasis and cirrhosis	FXR agonism restores ileal barrier function and reduces bacterial translocation	[25,43,44]

Abbreviations: FXR, farnesoid X receptor; SIBO, small-intestinal bacterial overgrowth.

Table S3—extended matrix of barrier, translocation and immune-activation biomarkers in cirrhosis.

Compartment reported	Biomarker	Specimen	Biological meaning	Reported value in cirrhosis	Ref.
Epithelial injury	I-FABP (FABP2)	Serum/plasma	Cytosolic enterocyte protein released on enterocyte damage	Marker of enterocyte injury and barrier failure	[24]
Epithelial injury	ZO-1	Serum/plasma	Tight-junction scaffolding protein	Tracks barrier injury in cirrhosis; prognostic value shown in bacteremic sepsis	[26,61]
Paracellular permeability	Zonulin-family peptides	Serum	Putative regulator of tight-junction permeability	Performed poorly across stages of cirrhosis in a head-to-head comparison; assay specificity remains a concern	[21]

Compartment reported	Biomarker	Specimen	Biological meaning	Reported value in cirrhosis	Ref.
Paracellular permeability	Differential sugar absorption (lactulose/mannitol), ^{51}Cr-EDTA	Urine	Functional measurement of intestinal permeability	Increased permeability tracks disease severity and predicts complications; reduced by non-selective beta-blockade	[24,69]
Microbial exposure	Lipopolysaccharide (endotoxin)	Plasma	Gram-negative cell-wall PAMP	Endotoxemia parallels the loss of occludin and claudin-1 and the severity of liver disease	[26,49]
Microbial exposure	LBP	Plasma	Hepatic acute-phase protein that binds endotoxin	Elevated in ascitic cirrhosis with marked immune and hemodynamic derangement; normalized by norfloxacin and reduced by non-selective beta-blockers	[49,69]
Translocation (direct)	Bacterial DNA	Blood and ascitic fluid	PCR detection of microbial DNA in normally sterile compartments	Detected in approximately one third of patients with culture-negative, non-neutrocytic ascites; *E. coli* predominant, with sequence identity indicating single-clone episodes	[45]
Innate/monocyte activation	sCD14 and presepsin	Plasma	Shed from monocytes upon LPS–TLR4 engagement	Presepsin performs comparably to established infection markers and independently predicts short-term mortality	[60]
Intestinal inflammation	Fecal calprotectin	Stool	Neutrophil-derived S100A8/A9 heterodimer	Rises with the severity of liver disease; screening tool for hepatic encephalopathy and spontaneous bacterial peritonitis	[62]
Systemic immune activation	suPAR	Plasma and ascites	Cleaved from membrane uPAR on immune-cell activation	Compartmentally regulated in decompensated cirrhosis; predicts short-term mortality	[63]
Systemic inflammation	IL-6, TNF-α, IL-1β, C-reactive protein, white-cell count	Plasma	Cytokine and acute-phase output of the innate compartment	Severity of systemic inflammation characterizes decompensated cirrhosis and tracks the course of acute-on-chronic liver failure; IL-6 falls with non-selective beta-blockade	[55,69]
Gut–vascular barrier	PV1 (plasmalemma vesicle-associated protein)	Plasma	Endothelial marker of gut–vascular barrier damage	Predicted transplant-free survival across stages of cirrhosis	[21]
Microbiome	Cirrhosis Dysbiosis Ratio	Stool	Ratio of autochthonous to non-autochthonous taxa	Falls with decompensation and infection; predicts organ failure and death	[30]

Abbreviations: I-FABP, intestinal fatty-acid-binding protein; IL, interleukin; LBP, lipopolysaccharide-binding protein; LPS, lipopolysaccharide; PAMP, pathogen-associated molecular pattern; PCR, polymerase chain reaction; PV1, plasmalemma vesicle-associated protein; sCD14, soluble CD14; suPAR, soluble urokinase plasminogen activator receptor; TLR4, Toll-like receptor 4; TNF-α, tumor necrosis factor-alpha; uPAR, urokinase plasminogen activator receptor; ZO-1, zonula occludens-1.

Table S4. Detailed evidence for interventions targeting the gut–liver axis in cirrhosis.

Intervention	Network node targeted	Study design and population	Principal findings	Evidence / current status	Ref.
Non-selective beta-blockers	Portal pressure; epithelial permeability; translocation	Prospective human study in cirrhosis with portal hypertension; complemented by experimental cirrhosis	Decreased gastroduodenal and intestinal permeability with lower LBP and IL-6, observed even in hemodynamic non-responders and associated with reduced variceal-bleeding risk; β-adrenergic overactivity contributes to barrier disruption and β-blockade restores barrier function and reduces translocation	Human prospective plus preclinical mechanistic; established for portal hypertension	[69,108]
Rifaximin	Microbial input – ammoniagenic and pathogenic flora	Randomized controlled trial in patients in remission from recurrent hepatic encephalopathy	Reduced breakthrough hepatic encephalopathy and hepatic-encephalopathy-related hospitalization	High – randomized evidence; a key component of current hepatic encephalopathy guidelines	[73,84]
Rifaximin – resistance safety	Antimicrobial resistance	Global multicenter inpatient cirrhosis cohort	No association between rifaximin exposure and drug-resistant infections	Observational; reassuring but not definitive	[89]
Norfloxacin, primary prophylaxis	Selective intestinal decontamination	Randomized controlled trial in low-protein ascites with advanced disease	Reduced incidence of spontaneous bacterial peritonitis, delayed hepatorenal syndrome and improved survival	High – randomized evidence; restricted to guideline-defined high-risk indications because of multidrug resistance	[68,88]
Fecal microbiota transplantation	Microbial input – microbiome restoration	Randomized clinical trial with a rationally selected donor in recurrent hepatic encephalopathy	Safe, with fewer hospitalizations and improved cognition and dysbiosis compared with standard care	Moderate – small randomized trials; investigational	[90]
Encapsulated and enema microbiota transplant	Microbial input – microbiome restoration	Randomized, placebo-controlled trial on a background of lactulose and rifaximin (THEMATIC)	Extends the safety and efficacy evidence base for microbiota transplantation in hepatic encephalopathy	Moderate – investigational	[94]
Probiotics and synbiotics	Microbial input; translocation markers	Heterogeneous randomized and observational studies	Reduced translocation markers and improved hepatic encephalopathy in some studies	Low to moderate – heterogeneity of formulations and endpoints limits firm conclusions	[3]
FXR agonist (obeticholic acid)	Epithelial compartment; antibacterial defense; gut–vascular barrier	Cholestatic and cirrhotic rodent models	Restored ileal barrier function and reduced bacterial translocation; FXR modulates the entry sites for translocation at the gut–vascular barrier	Preclinical – mechanistically attractive, not yet clinical for this indication	[25,43,44]
Long-term human albumin	Systemic innate response and endothelial stability	Open-label randomized trial in decompensated cirrhosis with ascites (ANSWER)	Prolonged survival and acted as a disease-modifying treatment	Strong – randomized evidence	[109]
Nutrition; avoidance of unnecessary proton-pump inhibitors	Microbial input – small-intestinal bacterial overgrowth	Observational and physiological studies	Acid suppression promotes small-intestinal bacterial overgrowth; adequate nutrition and correction of micronutrient deficiency support barrier function	Low to moderate – supportive care	[34]

Intervention	Network node targeted	Study design and population	Principal findings	Evidence / current status	Ref.
Transjugular intrahepatic portosystemic shunt	Portal pressure; barrier and immune dysfunction	Prospective human study of portal decompression	Reversal of markers of gut-barrier dysfunction and cirrhosis-associated immune dysfunction	Moderate – prospective human data; supports partial reversibility	[58]
Enterosorbents, endotoxin-neutralizing agents, next-generation microbiome therapeutics	Luminal PAMP load; containment of translocation	Early-phase development	Bind bacterial toxins without altering bacterial growth kinetics, reflecting a shift from suppressing the microbiota towards restoring the barrier	Preclinical to early clinical	[11]

Abbreviations: FXR, farnesoid X receptor; IL-6, interleukin-6; LBP, lipopolysaccharide-binding protein; PAMP, pathogen-associated molecular pattern.

Table S5. Principal cells and mediators of inter-compartment crosstalk at the gut–liver interface.

Source compartment	Cell type	Mediator(s)	Target compartment	Signal type	Consequence in cirrhosis	Evidence type	Ref.
Epithelial / barrier	Enterocyte	TSLP, IL-25, IL-33; chemokines	Innate and adaptive	Activating / instructive	Signaling is biased towards a pro-inflammatory tone as junctional integrity is lost	Animal and human in vitro; inferred for cirrhosis	[5,6]
Epithelial / barrier	Paneth cell	α-defensins, lysozyme, Reg3 lectins	Luminal microbiota	Antimicrobial	Reduced output with loss of bacterial–mucosal segregation	Human in vivo and animal	[3,25]
Epithelial / barrier	Goblet cell	MUC2 mucin	Luminal microbiota	Physical / chemical	Mucus-layer thinning	Human in vivo and animal	[3]
Innate immune	Lamina propria macrophages and dendritic cells	TNF-α, IL-6, IL-1β, IL-23	Epithelial and adaptive	Activating; feed-forward	Sustains systemic inflammation and further degrades the barrier	Human in vivo (systemic) and animal (mucosal)	[10,55]
Innate immune	Group 3 innate lymphoid cell (ILC3)	IL-22	Epithelial	Homeostatic feedback	Reparative signal becomes insufficient as disease advances	Animal; inferred in human cirrhosis	[27]
Innate immune	Kupffer cell	TNF-α, IL-6, reactive oxygen species	Systemic	Activating	Distal node of a single epithelial–myeloid–Kupffer axis fed by portal blood	Human in vivo and animal	[3,11,12]
Innate immune	Circulating monocyte (with hepatic LBP)	sCD14 and presepsin; LBP	Systemic (read-out)	Measurable index	Provides quantifiable readouts of innate activation	Human in vivo	[49,60]
Adaptive effector	Th17 cell	IL-17, IL-22	Epithelial	Feedback – reparative at physiological levels, injurious when sustained	Expansion at the expense of regulatory T cells, closing a loop onto the barrier	Human in vivo (circulating) and animal (mucosal)	[6,28]
Adaptive effector	Regulatory T cell	IL-10, TGF-β	Innate and epithelial	Regulatory	Reduced regulatory tone	Human in vivo (peripheral); animal (mucosal)	[28]

Source compartment	Cell type	Mediator(s)	Target compartment	Signal type	Consequence in cirrhosis	Evidence type	Ref.
Adaptive effector	IgA plasma cell	Secretory IgA	Luminal microbiota	Confinement	Impaired generation and transepithelial delivery of sIgA	Human in vivo and animal	[3,10]
Adaptive effector	MAIT and other unconventional T cells	IFN-γ, TNF-α, granzyme	Innate and epithelial	Rapid, innate-like antibacterial	Chronic engagement by translocating bacteria	Human in vivo	[10,12]
Stromal–endothelial	Subepithelial myofibroblast; enteric glia	Prostaglandins, growth factors, neurotrophic mediators	Epithelial	Supportive/reparative	Modulate local inflammatory and reparative signaling	Animal and in vitro; inferred	[4]
Stromal–endothelial	Gut–vascular barrier endothelium	Wnt/β-catenin signaling; PV1	Portal circulation	Gatekeeping	Reduced β-catenin signaling and increased PV1 permit systemic dissemination of bacteria and PAMPs	Animal and human in vivo	[4,44]
Stromal–endothelial	Intestinal blood-vessel-associated macrophage	—	Gut–vascular barrier	Regulatory	Dysfunction contributes to gut–vascular barrier breakdown in human cirrhosis	Animal and human in vivo	[20]
Luminal input/output	Translocating bacteria and PAMPs	LPS, bacterial DNA, peptidoglycan, muramyl dipeptide	All four compartments	Input and output	Re-enters the network at the top, generating self-amplifying feed-forward loops	Human in vivo and animal	[7,9]

Abbreviations: IFN-γ, interferon-gamma; IL, interleukin; ILC3, group 3 innate lymphoid cell; LBP, lipopolysaccharide-binding protein; LPS, lipopolysaccharide; MAIT, mucosa-associated invariant T cell; MUC2, mucin-2; PAMP, pathogen-associated molecular pattern; PV1, plasmalemma vesicle-associated protein; Reg3, regenerating islet-derived protein 3; sCD14, soluble CD14; sIgA, secretory immunoglobulin A; TGF-β, transforming growth factor-beta; Th17, T-helper-17 cell; TNF-α, tumor necrosis factor-alpha; TSLP, thymic stromal lymphopoietin.

Table S6. Full electronic search strings by database (search completed 31 August 2026).

Database/source	Search string
PubMed/MEDLINE	("liver cirrhosis"[MeSH] OR cirrho*[tiab]) AND ("intestinal barrier"[tiab] OR "tight junction*"[tiab] OR "intestinal permeability"[tiab] OR "bacterial translocation"[MeSH] OR "gut-vascular barrier"[tiab] OR dysbiosis[tiab] OR "small intestinal bacterial overgrowth"[tiab] OR endotoxemia[tiab] OR lipopolysaccharide*[tiab] OR "mucosal immunity"[tiab] OR "innate lymphoid"[tiab] OR Th17[tiab] OR "mucosa-associated invariant T"[tiab] OR "Kupffer cell*"[tiab] OR "cellular crosstalk"[tiab] OR "cirrhosis-associated immune dysfunction"[tiab] OR "acute-on-chronic liver failure"[tiab] OR "spontaneous bacterial peritonitis"[tiab] OR "hepatocellular carcinoma"[tiab]) AND (english[lang])
Embase (Ovid)	(liver cirrhosis/ OR cirrho*.ti, ab.) AND (intestinal barrier. ti, ab. OR tight junction*.ti, ab. OR intestinal permeability/ OR bacterial translocation/ OR gut-vascular barrier. ti, ab. OR dysbiosis.ti,ab. OR small intestinal bacterial overgrowth/ OR endotoxemia/ OR lipopolysaccharide/ OR mucosal immunity/ OR innate lymphoid cell*.ti, ab. OR Th17.ti,ab. OR mucosal associated invariant T cell/ OR Kupffer cell/ OR cellular crosstalk. ti, ab. OR cirrhosis-associated immune dysfunction. ti, ab. OR acute on chronic liver failure/ OR spontaneous bacterial peritonitis/ OR liver cell carcinoma/) AND english.lg. Limit to (human and English language)
Web of Science Core Collection	TS=(cirrho*) AND TS=("intestinal barrier" OR "tight junction*" OR "intestinal permeability" OR "bacterial translocation" OR "gut-vascular barrier" OR dysbiosis OR "small intestinal bacterial overgrowth" OR endotoxemia OR lipopolysaccharide* OR "mucosal immunity" OR "innate lymphoid" OR Th17 OR "mucosa-associated invariant T" OR "Kupffer cell*" OR "cellular crosstalk" OR "cirrhosis-associated immune dysfunction" OR "acute-on-chronic liver failure" OR "spontaneous bacterial peritonitis" OR "hepatocellular carcinoma"); language: English
Intervention terms (all databases, combined with the above)	rifaximin OR norfloxacin OR probiotic* OR synbiotic* OR "fecal microbiota transplant*" OR "fecal microbiota transplant*" OR "farnesoid X receptor" OR "FXR agonist*" OR "non-selective beta-blocker*" OR albumin OR "sodium-glucose cotransporter*" OR SGLT2 OR enterosorbent* OR "defined consorti*."
Registries	ClinicalTrials.gov and WHO ICTRP searched for: (cirrhosis OR "hepatic encephalopathy") AND (transplant OR rifaximin OR SGLT2 OR microbiome); records screened for adequately powered trials with clinical endpoints.
