# Supplementary material for: Cellular Crosstalk at the Gut–Liver Interface in Liver Cirrhosis: Epithelial Barrier Failure, Bacterial Translocation and Immune Activation as a Bidirectional Network
Source: Cells. 2026 Sep 23;15(19):1739. doi: 10.3390/cells15191739 (PMC13664048; doi:10.3390/cells15191739)
Supplement: Supplementary file 1 [file cells-15-01739-s001.zip › File S1.pdf]

Supplementary File S1. Completed SANRA checklist

The Scale for the Assessment of Narrative Review Articles (SANRA) comprises six items, each scored 0 (low), 1 (medium) or 2 (high), giving a maximum total of 12 [1]. The table below reports the score the authors assigned to the present review for each item, the section of the main manuscript in which the item is addressed, and a brief explanatory note. Scoring represents the authors' self-assessment against the published SANRA anchor definitions [1] and was not performed by independent raters. Section numbers refer to the revised main manuscript.

Item	SANRA item	Score (0–2)	Where addressed	Explanatory note
1	Justification of the article's importance for the readership	2	Abstract; Section 1 (Introduction); Section 1.1	The Introduction sets out the global burden of cirrhosis and the central role of the gut–liver axis in its systemic complications, explains why the prevailing linear (upstream-to-downstream) model is insufficient, and Section 1.1 positions the review against eight existing accounts (Table 1) to state what it adds: a cirrhosis-specific, stage-resolved, bidirectional framework incorporating human evidence published in 2024–2026.
2	Statement of concrete aims or formulation of questions	2	Abstract; Section 1 (final paragraph); Section 1.1	The review states its aim of integrating dysbiosis, barrier failure, translocation and immune activation into a single four-compartment framework and lists the four specific choices that define its scope (cirrhosis-specific and stage-resolved treatment; bidirectionality as the organizing axis; integration of 2024–2026 human data; provenance labelling of every mechanistic claim). It also states explicitly what the review does not attempt to cover.
3	Description of the literature search	2	Section 2.1 (Search Strategy); Section 2.2 (Eligibility); Supplementary Table S6	Databases (PubMed/MEDLINE, Embase via Ovid, Web of Science Core Collection), coverage (database inception to 31 August 2026), the search-term groups, hand-searching of reference lists, and searches of ClinicalTrials.gov and the WHO ICTRP are described; the complete search strings for each database are given in Supplementary Table S6. Inclusion and exclusion criteria (language, population, study types) are stated in Section 2.2. Section 2.4 and Section 12 state the limits of the search (not registered, not performed in duplicate, no formal record counts or flow diagram).
4	Referencing	2	Throughout the main text; Tables 1–3; Supplementary Tables S1–S5; reference list (123	Key statements are supported by references to primary studies, systematic reviews/meta-analyses or clinical practice guidelines. Review articles are used only for conceptual framing and are not cited as sources of primary data (Section 2.3). Findings available only as conference

Item	SANRA item	Score (0–2)	Where addressed	Explanatory note
			references, each with DOI)	abstracts or preprints are identified as such at the point of citation.
5	Scientific reasoning	2	Section 2.3 (Prioritization of Evidence); Sections 3–10; Supplementary Table S5	Evidence is prioritized in a pre-specified order (randomized controlled trials and their meta-analyses; prospective human cohorts and human-tissue mechanistic studies; retrospective cohorts; animal and in vitro studies). The provenance of each mechanistic claim (human, animal, in vitro or inferred) is labelled in the text and in Supplementary Table S5; conflicts between randomized and observational evidence are presented rather than resolved; associative human data are distinguished from causal inference; and the strength of evidence for each intervention is graded (Table 3, Figure 4).
6	Appropriate presentation of data	2	Sections 4–6, 8–10; Tables 2 and 3; Supplementary Tables S3 and S4	Where the underlying studies permit, endpoint data are presented with absolute event rates and effect sizes with 95% confidence intervals (e.g., hazard ratio for mortality associated with serum bacterial DNA; odds ratio for hepatic encephalopathy recurrence after fecal microbiota transplantation; hazard ratio for acute-on-chronic liver failure in the simvastatin–rifaximin trial), pooled prevalence estimates for small-intestinal bacterial overgrowth are given with their comparators, and biomarkers are presented by context of use (mechanistic, diagnostic, prognostic, predictive) with their limitations.
**Total score**		**12 / 12**	Authors' self-assessment; see note above.	

SANRA scoring anchors used [1]

Item	SANRA item	Anchor definitions (0 / 1 / 2)
1	Justification of the article’s importance for the readership	0 = importance not justified; 1 = importance justified in part; 2 = importance fully justified for the intended readership.
2	Statement of concrete aims or formulation of questions	0 = no aims or questions stated; 1 = aims formulated only in general terms; 2 = concrete aims or questions formulated.
3	Description of the literature search	0 = search not described; 1 = search described in part (e.g., only databases or only search terms); 2 = search strategy presented (databases, search terms, inclusion criteria, time frame).
4	Referencing	0 = key statements not supported by references; 1 = key statements supported in part; 2 = key statements supported by references.
5	Scientific reasoning	0 = key statements not supported by appropriate evidence; 1 = supported in part; 2 = supported by appropriate evidence (e.g., randomized controlled trials for treatment questions) with appropriate reasoning.
6	Appropriate presentation of data	0 = relevant endpoint data not presented; 1 = presented in part; 2 = relevant endpoint data presented appropriately (e.g., absolute and relative risks, effect sizes with confidence intervals).

Reference

1. Baethge, C.; Goldbeck-Wood, S.; Mertens, S. SANRA—a scale for the quality assessment of narrative review articles. Res. Integr. Peer Rev. 2019, 4, 5. https://doi.org/10.1186/s41073-019-0064-8.
